# Central and peripheral pathogenetic forms of type 2 diabetes: a proof-of-concept study

**DOI:** 10.1530/EC-16-0009

**Published:** 2016-02-04

**Authors:** Dmitry M Davydov, Malik K Nurbekov

**Affiliations:** 1 Laboratory of Neuroimmunopathology, Institute of General Pathology and Pathophysiology, Russian Academy of Medical Sciences, Moscow, Russia; 2 Laboratory of Sociogenomics, Moscow State Pedagogical University, Moscow, Russia

**Keywords:** fasting plasma glucose, glycated haemoglobin, gene polymorphism, *DRD2/ANKK1*, *PGC1A*, *ACE*, central arousal, muscle activity, vascular resistance, type 2 diabetes

## Abstract

**Hypothesis:**

Previous studies provide evidence that glycated haemoglobin (HbA_1c_) and fasting plasma glucose (FPG) should not be considered as interchangeable alternatives in the diagnosis of the same type 2 diabetes, but as indicators of its different pathogenetic subtypes. This study was conducted to determine whether a particularly high amount of glucose in either HbA_1c_ form or in fasting plasma would be found in diabetic patients genetically predisposed for either intensive cognitive or intensive muscle metabolic activity, respectively.

**Methods:**

HbA_1c_ and FPG levels, polymorphisms of genes indicating the predisposition to different cognitive activity (the dopamine D2 receptor (*DRD2/ANKK1*)), muscle activity (peroxisome proliferator-activated receptor gamma, coactivator 1 alpha (*PGC1A*(*PPARGC1A*))), and vascular regulation of general metabolic activity (the angiotensin 1 converting enzyme (*ACE*)) were assessed in diabetic patients and nondiabetic controls.

**Results:**

*DRD2/ANKK1* polymorphism that affects baseline central arousal determined HbA_1c_ variations uncorrelated with FPG in total and clinical groups. The mutation of PGC1A mainly affecting peripheral glucose metabolism had an effect on FPG correlated or uncorrelated with HbA_1c_ depending on the effect assessment in the total sample or in the nondiabetic group, respectively. *ACE* insertion/deletion (I/D) gene polymorphism was associated with both HbA_1c_ and FPG fluctuations, but only in diabetic patients.

**Conclusion:**

The findings provide evidence that the HbA_1c_ and FPG may predict the risks for different subtypes of type 2 diabetes associated with either brain or muscle metabolic activity in genetically vulnerable people.

## Introduction

Despite the known limitations of diagnostic criteria and early risk for type 2 diabetes derived from fasting (FPG) and/or 2-h plasma glucose levels, they are still widely accepted. Glycated hemoglobin A1 or the percentage of total hemoglobin that is bound by glucose (HbA_1c_) reflects an average level of plasma glucose over the previous 2–4 months and is also widely used as a marker of chronic glycemia. It was concluded that HbA_1c_ and FPG display a different sensitivity versus specificity in identifying people at risk for later development of diabetes, and Expert Committees in diabetes consider that a better biochemical marker should be selected as a more reliable diagnostic tool (1, 2). However, this discordance may occur because HbA_1c_ and FPG each measure different physiological processes associated with the risk for type 2 diabetes. In this case, these glycemic parameters should be considered as partly overlapped, but not as interchangeable alternatives.

Previous studies showed that HbA_1c_ is not affected by plasma glucose instability and has a close relationship with mean or average plasma glucose (MPG) (3). However, correlations between HbA_1c_ and FPG are low. Twin, family, and genome-wide association studies show that HbA_1c_ and FPG levels are heritable with substantial genetic effects on their inter-individual variations, and that genetic factors influencing them do not overlap (4). Although some heritable HbA_1c_ variations are unrelated to glycemic status in individuals with and without type 2 diabetes (e.g., altered erythrocyte physiology), individual variance in both glycemic parameters can reflect different aspects of energy metabolism that are modulated by different gene polymorphisms. However, hypothesis-free, genome-wide association studies with multiple testing do not seem very helpful in exploring this challenge because they may miss some important physiological relationships (5). The main analytical approach of genome-wide association studies is focused on minimizing the false positive rate at the expense of controlling the false negative rate, and this bias determines the ‘missing heritability’ of type 2 diabetes susceptibility (6, 7, 8). This gives an additional warning that results of data-driven studies should not exclusively dictate or restrict scientific development from using the conventional approach based on prior hypotheses (9, 10).

The hypothesis-driven candidate gene approach based on an inferred physiological role of previously discovered genes may investigate the subject more effectively. For example, physiological mechanisms between feeding and fasting are differently regulated for keeping glucose levels sufficient: (i) for permanent functioning of the brain and other glucose-dependent tissues (switching between idled and non-idled brain conditions; neutral, positive, and negative moods; and various cognitive activities) and (ii) for an intermittent energy supply to all other organs (e.g., switching between glucose and fatty acid oxidation in active and relaxed muscles) (11). Previous findings allow us to consider that HbA_1c_ level may be associated with genetic factors influencing more stable central mechanisms (habitual cognitive and dietary patterns), but FPG level may be determined by genetic factors influencing more flexible peripheral mechanisms which regulate energy metabolism (cardiovascular and metabolic activity patterns). For example, a study of children with and without attention-deficit/hyperactivity disorder (ADHD) found that FPG did not differ between the groups, but HbA_1c_ was higher in the ADHD-group (12). This HbA_1c_-related glucose regulation may be modulated by a central dopaminergic system in general and by polymorphism of the dopamine D2 receptor (*DRD2*) gene in particular (e.g., rs1800497 (*TaqIA*) single-nucleotide polymorphism (SNP), later identified within the exon 8 of ankyrin repeat and kinase domain containing 1 (*ANKK1*)). Mutations in this gene were found to be associated with ADHD and appear to be relevant to cognitive skills, as well as type 2 diabetes, dietary, and other motivated and compulsive/impulsive behaviors (13, 14, 15, 16). Other studies support that MPG- or HbA_1c_-, but not FPG, -related glucose regulation is associated with cognitive performance and dietary preferences.

For example, only higher HbA_1c_ or MPG was associated with cognitive decline and cognitive impairments (e.g., lower scores of visual motor speed, attention, learning, and verbal memory) in people with and without type 2 diabetes (17, 18). These cross-sectional and prospective relationships were not mediated by cardiovascular (CV) or other metabolic complications and may be determined by more fat, carbohydrate, or generally higher caloric food preference as habitual dietary behavior in people with higher HbA_1c_ (19, 20, 21). It seems evolutionary justified, that meal restriction claims better performance skills for obtaining food, but an easy access to plenty of meal results in a ‘lazy’ or ‘idled’ brain (i.e., in the brain with low cognitive performance). In these cases, the levels of HbA_1c_ may display an amount of glucose unclaimed by the brain and irrevocably removed from energy metabolism. A particularly high amount of this unclaimed glucose in HbA_1c_ form may be predicted in people with the phenotypically ‘idled’ or ‘lazy’ brains that are genetically predisposed for hard cognitive working (e.g., individuals with *A1-T* allele of *TaqIA*). Fewer studies found that higher FPG values were also associated with lower cognitive skills, but only in patients with CV diseases or metabolic syndrome (22, 23). Compared with HbA_1c_, the FPG relationship with cognitive functions may be driven indirectly by peripheral hyperglycemia-related processes resulting in severe cardiovascular and metabolic impairments (24).

In some studies, the presence of *D* allele in case of the angiotensin 1 converting enzyme (*ACE*) insertion/deletion (*I/D*) gene polymorphism (determining higher ACE levels and thus higher vascular resistance) conferred an increased risk of glucose intolerance (detected by higher 2-h glycemia) in a nondiabetic population (25). However, this association may be moderated by other genetic or nongenetic factors, as another study found that overweight women who are homozygous for the *D* allele of this gene had more intensive glucose utilization (detected by hyperinsulinemic–euglycemic clamps) compared with those who are homozygous for the *I* allele (26). *ACE* gene polymorphism, as well as gene polymorphisms affecting other metabolic factors like peroxisome proliferator-activated receptor gamma (PPARG2) and its coactivator (PGC1A (*PPARGC1A*)), may have opposite effects on glucose metabolism in different (e.g., clinical and nonclinical) populations (27, 28, 29, 30, 31). Hemodynamic (*ACE*) and metabolic (*PPARG2* and PGC1A) gene polymorphisms were found to have a weak impact on HbA_1c_ level and cognitive functions, but have a strong impact on body composition and physical activity in the general population and diabetic patients (32, 33, 34, 35, 36). FPG, but not HbA_1c_, is mainly related to body composition (e.g., body weight, visceral fat area, body mass index, body fat percentage, body muscle percentage, waist circumference) and exercise (walking, steps/day and physical activity, min/day) indicators (20). It seems physiologically justified, when ‘lazy’ or ‘idled’ muscles with low metabolism do not claim the glucose intended for them, its unutilized and undeposited excess is detected in fasting and 2-h plasma. A particularly high amount of unclaimed glucose in fasting plasma may be predicted in persons with the ‘lazy’ muscles that are genetically predisposed for intensive or hard physical work (e.g., individuals with *D* allele of *ACE I/D* gene polymorphism or *G* allele of rs8192678 (*Gly482Ser*) polymorphism of *PGC1A* gene).

This research addresses two above-mentioned predictions of central and peripheral physiological mechanisms associated with HbA_1c_ and FPG and thus different central and peripheral pathophysiological forms of type 2 diabetes by studying the relationships between polymorphisms of central (*DRD2*), hemodynamic (*ACE*), and metabolic (*PGC1A*) genes and these two glycemic indicators in diabetic patients and nondiabetic control group.

## Subjects and methods

### Ethical statement

Institutional and governmental regulations concerning the ethical use of human volunteers were followed. The study was approved by the Ethics Committee of the Sholokhov Moscow State University for the Humanities and performed in compliance with the recommendations of Helsinki Declaration II.

### Study populations

This study was based on a larger cohort of outpatients diagnosed with type 2 diabetes, living in Moscow (Russia), and listed in a local medical register. For the study, 103 participants (73 females) aged 23–83 years (mean ± s.d., 65.7 ± 11.2 years) with undefined ethnicity (due to ethical regulations of the local medical register) were randomly selected from a sub-group of patients with uncomplicated type 2 diabetes for no less than 2 years. Classification of type 2 diabetes was done according to the WHO diagnostic criteria (37). For inclusion in the study, the patients needed to be a noncompliant with the treatment regimen in order to decrease the confounding effect of the treatment on expected relationships. Of the total sample of diabetic patients listed in the local medical register, 50% were identified as missing medication in the previous week. The recommended treatment modalities in the selected sample were metformin alone (20%); metformin in combination with dipeptidyl peptidase IV inhibitor (5%); glucagon-like peptide 1 alone (5%); and insulin alone (10%) or in combination with oral antidiabetic agents (metformin and sulfonylurea; 60%). Sixty subjects (42 females) without diabetes and undefined ethnicity (due to the same ethical regulations) aged 19–92 years (53.8 ± 16.2 years) were enrolled in the control group. They visited primary care centers for a regular annual medical examination and did not either have hypertension, coronary heart disease, or any other neuropsychiatric, cardiovascular, or metabolic diseases. All selected patients and healthy subjects signed a written consent for participation in the study.

An effective sample size for genetic model with continuous outcomes was calculated using Quanto (38). Sample size = 60 was found effective for *α* = 0.05 and power = 0.80 with *β* = 0.60, dominant allele frequency = 0.54 and mean (s.d.) of FPG = 5.3 (0.7) mmol/L obtained from a previous study and accepted for a group of subjects without diabetes (39). Additional 43 subjects were included in the patient group to cover for possible allele frequency deviation associated with the disease (40).

### Laboratory assays

Blood samples of the people with and without diabetes were collected during 2012–2015 on a regular weekday morning after an overnight fast during an outpatient visit to local hospital laboratories or primary care centers in Moscow (Russia) and immediately delivered to the Moscow Diagnostic and Clinical Center #1. The samples were analyzed for fasting plasma glucose (FPG), glycated hemoglobin A1 (HbA_1c_), total cholesterol, low- and high-density lipoprotein-cholesterol (direct measurements), triglycerides, plasma creatinine, total bilirubin, total protein, aspartate transaminase, and alanine transaminase by standard laboratory methods using commercially available kits (Olympus and Siemens, Germany; BioSystems S.A., Barcelona, Spain) and automatic analyzers (ADVIA 2400 Clinical Chemistry System, Siemens Healthcare GmbH, Erlangen, Germany and Olympus AU5800/2700, Olympus Diagnostica GmbH, Hamburg, Germany).

### Genetic analysis

Total genomic DNA was extracted from peripheral blood leukocytes and assessed as described in detail elsewhere (41). In particular, SNP genotyping of *PGC1A* (*Gly482Ser*, rs8192678, *G > A* nucleotide change) and *DRD2/ANKK1* (*TaqIA*, rs18000497, *A1-T,* and *A2-C* nucleotide change) was performed by the real-time PCR method using a TaqMan (hydrolysis) probe-based assay (Thermocycler DT-322; DNA-Technology, Russia) or using 2% agarose gels with a 100-bp DNA Ladder (SibEnzyme Ltd, Russia) and a gel imaging system for genotyping *ACE* (insertion/deletion, *I/D*) polymorphisms in intron 16 (42). The end-point readings were analyzed according to the manufacturer’s instructions. One-third of the samples of patients and healthy subjects was randomly processed in duplicate for each genotype analysis to protect the study against genotyping errors. Genotype data were obtained in 98.7–100% of the DNA samples with a genotype error rate of 0.0%.

### Data analyses

Descriptive and inferential analyses were performed by SPSS (SPSS Science, Chicago, IL) using Pearson product–moment correlation and General Linear Models by Type III method (GLM) to evaluate significance of multivariate and univariate effects and bootstrapped confidential intervals (CIs) of regression estimates. Bias-corrected and accelerated bootstrap procedure with 1000 bootstrap replications of regression coefficients was used to derive their nonparametric 95% CIs from empirical sampling distribution. The bootstrap procedure was considered as a robust alternative to inference based on parametric assumptions (such as normally distributed errors) to confirm or validate findings obtained by parametric analyses, and is recommended for reporting inferences in scientific reports (43). Differences at *P* < 0.05 were regarded as significant for parametric analyses. Parameter estimates are expressed as nonstandardized (B) regression coefficients and their standard errors (S.E.) or as means (M) and their standard deviations (s.d.). Where necessary, a partial η^2^ was reported as a measure of strength of association (effect size), which is comparable to R^2^ expressing the percentage of explained variance. Sex and age were included in all models to adjust effects for these factors. As the distribution of age was skewed, its common logarithm (log_10_) was used.

The GLM procedure was conducted with two measures of glycemia, FPG and HbA_1c_, treated as continuous dependent variables representing multiple measurements of the same process to test the hypothesis that both together are affected by gene polymorphisms. All multivariate *F* values were obtained by Pillai’s trace statistic, which is equivalent to partial η^2^ and R^2^ measures of effect size, and tolerant of the violation of homogeneity of variance–covariance matrices. If the overall multivariate test achieved (<0.05) or approached significance (<0.10) for a particular gene polymorphism effect, the regression estimates of each of the two glycemic measures were inspected to identify the specific dependent variable (FPG or/and HbA_1c_) that contributed to the overall effect. Final inferences of significance of the effects and relationships were based on results of the bootstrap nonparametric procedure. Differences in proportions and Hardy–Weinberg equilibrium (HWE) were assessed by Fisher’s exact test.

## Results

Table 1 lists the values of demographic and metabolic parameters separately for patients with diabetes and control participants. Patients in diabetes group were significantly older and had significantly higher values of FPG, HbA_1c_, triglycerides, and alanine transaminase, but lower values of total and high-density lipoprotein cholesterols than controls. FPG weakly correlated with HbA_1c_ in diabetes and control groups (*r* = 0.35 and 0.22, *P* < 0.001 and 0.10, respectively). There were no significant differences among the groups in sex, creatinine, total protein, low-density lipoprotein cholesterol, aspartate transaminase, and total bilirubin.

**Table 1:** Demographic and laboratory characteristics of the samples^a^

**Groups:**	**Diabetes**	**Control**
Characteristics	Mean (s.d.)	Mean (s.d.)
N	103	60
Sex, male/female	30/73	18/42
Age (years)	65.7 (11.2)^b^	53.8 (16.2)
Fasting plasma glucose (FPG, mmol/L)	8.99 (3.08)^b^	4.76 (0.59)
Glycated hemoglobin (HbA_1c_, %)	8.4 (3.9)^b^	5.3 (2.6)
Glycated hemoglobin (HbA_1c_, mmol/mol)	68.1 (19.1)^b^	34.4 (4.5)
Creatinine (μmol/l)	101.3 (39.4)	97.9 (11.5)
Total protein (g/L)	71.4 (4.2)	73.1 (2.7)
Total cholesterol (Chol, mmol/L)	5.24 (1.05)^b^	5.94 (1.40)
Triglyceride (TG, mmol/L)	2.15 (1.48)^d^	1.74 (0.85)
High-density lipoprotein (HDL, mmol/L)	1.26 (0.30)^d^	1.36 (0.37)
Low-density lipoprotein (LDL, mmol/L)	2.84 (0.81)	2.78 (0.96)
Alanine transaminase (ALT, IU/L)	29.7 (20.8)^c^	20.7 (9.0)
Aspartate transaminase (AST, IU/L)	24.8 (11.1)	23.8 (5.5)
Total bilirubin (μmol/l)	11.2 (4.8)	11.8 (5.2)

a Means and comparisons; comparisons for metabolic data are presented after adjustment for age and sex (analysis of covariance).

b *P *< 0.001;

c *P *< 0.005;

d *P *< 0.05.

### Hardy–Weinberg equilibrium test

Distribution of *DRD2/ANKK1* genotype in diabetes group, *ACE* and *PGC1A* genotypes in control group (*P* < 0.01, 0.005, and 0.05, respectively), and all three genetic loci in the total sample (*P* < 0.05) was significantly different from the expectations of HWE. Although, in principle, genotyping errors may be responsible for the observed statistically significant deviations from HWE, this was unlikely for these samples because double scoring and duplicate genotyping in randomly selected subsets of 30% individuals confirmed the results for each locus. Therefore, the probable reason for the departure of observed genotype frequencies for the three loci is not genotyping error but the association of loci with disease and/or population stratification (e.g., intensive multiethnic migration, generations overlapping, and unequal sex ratio (M < F) in this region). More importantly, in contrast to case–control (i.e., gene–disease association) studies, the expected inferences on quantitative association of the polymorphisms with FPG and HbA_1c_ phenotypes or its lack should remain valid and be independent of the fact that these polymorphisms in the type 2 diabetes or control individuals are not in HWE (44).

Significant simple and interaction effects of *ACE* and *PGC1A* polymorphisms and interaction effect of Sex*DRD2/ANKK1 genotype on age in the total sample confirmed the assumption of generations overlapping as a probable cause violating HWE in this study. The presence of the *PGC1A* gene *GG* genotype and the *ACE I* allele each separately increased the odds of surviving to older age (Fs(1,159) = 4.96 and 4.16, *P* = 0.027 and 0.043; η^2^ = 0.03 and 0.03, respectively, after adjustment for Sex; confirmed by bootstrap 95% CIs: 0.01–0.08 and 0.004–0.08, respectively). However, association of the presence of the *PGC1A A* allele with the *ACE* gene *DD* genotype significantly increased the risk of not surviving to older age (F(1,158) = 4.46, *P* = 0.036; η^2^ = 0.03, after adjustment for Sex; confirmed by bootstrap 95% CI: 0.01–0.15). Women with *DRD2/ANKK1* gene *A2A2* genotype and men with *DRD2/ANKK1 A1* allele had higher odds to survive to older age (F(1,157) = 4.37, *P* = 0.038; η^2^ = 0.03; confirmed by bootstrap 95% CI: 0.02–0.18).

### Genotype distribution

Compared with the control group, the frequency of the *G* allele polymorphism of *PGC1A* gene was significantly higher, but the frequency of the *PGC1A AA* genotype was lower in the diabetes group (Table 2). No evidence of differences between the groups was found in genotype or allele frequency of the other two genetic polymorphisms.

**Table 2:** Genotype frequencies of studied loci in patients with diabetes and controls without diabetes

**Groups:**	**Diabetes**	**Control**
Genetic loci	% (*N*) ^a^	% (*N*) ^a^
*DRD2/ANKK1 TaqIA* (C32806T, rs1800497) polymorphism		
*CC(A2A2)*	64 (66)	61 (36)
*CT(A2A1)*	26 (27)	34 (20)
*TT(A1A1)*	10 (10)	5 (3)
ACE (*I/D*) polymorphism		
*DD*	32 (33)	47 (28)
*ID*	41 (42)	28 (17)
*II*	27 (28)	25 (15)
PGC1A (Gly482Ser, rs8192678) polymorphism^b^		
*GG*	56 (58)	41 (24)
*GA*	36 (37)	35 (21)
*AA*	8 (8)	24 (14)

a Numbers of individuals vary between different loci due to missing genotypes in some subjects.

b Fisher’s exact *P* < 0.05 between the groups.

### Levels of FPG and HbA_1c_ in different genotypes

Multivariate test found that *DRD2/ANKK1* and *PGC1A*, but not *ACE* allele polymorphism, significantly determined the fluctuation of glycemic status in the total group (Pillai’s traces = 0.04 (0.03), 0.05 (0.04), and 0.01 (0.01); Fs(2) = 3.57 (2.75), 3.64 (3.03), and 0.61 (1.15); *P* = 0.030 (0.067), 0.028 (0.051), and 0.546 (0.321), respectively; data in square brackets here and further are unadjusted). HbA_1c_, but not FPG, levels were significantly higher in non-*A2* allele carriers of *DRD2/ANKK1* (*A1A1* genotype) compared with subjects with *A2A2* & *A2A1* genotypes, but both HbA_1c_ and FPG levels were higher in *G* allele carriers of *PGC1A* (*GG* & *GA* genotypes) compared with subjects with *AA* genotype (Table 3).

**Table 3:** Main effects of polymorphisms of the dopamine D2 receptor (*DRD2/ANKK1*), angiotensin 1 converting enzyme (*ACE*), and peroxisome proliferator-activated receptor gamma, coactivator 1 alpha (*PGC1A*) genes on glycemic parameters: fasting plasma glucose (FPG) and glycated hemoglobin *A1* (HbA_1c_) in the total group (univariate analyses)

		**Dependent variables**^b^			**Parameter estimates**^c^				
**Independent variables**		FPG (mmol/L)	HbA_1c_ (%)	HbA_1c_ (mmol/mol)	Total group (*N* = 163)				
Genes	Alleles ^a^	M (s.d.)	M (s.d.)	M (s.d.)	B (S.E.)	*t* (*P*)	*η*^2^	Bootstrap 95% CI	
Model 1									
*DRD2/ANKK1*	*A2* carriers	*7.52 (3.25)*	7.2 (4.2)	**54.7 (22.1)**	**−13.9 (6.38)**	**−2.17 (0.031)**	**0.03**	**−27.1**	**−1.63**
	non-*A2* carriers	*7.98 (2.99)*	8.4 (4.6)	**68.5 (27.2)**	**−***0.45 (0.93)*	**−***0.49 (0.626)*	*0.00*	**−***2.09*	*1.07*
Model 2									
*ACE*	*D* carriers	*7.77 (3.45)*	7.4 (4.3)	**56.9 (23.6)**	**5.13 (3.92)**	**1.31 (0.193)**	**0.01**	**−2.81**	**12.52**
	non-*D* carriers	*6.94 (2.42)*	6.9 (3.9)	**51.8 (18.8)**	*0.83 (0.57)*	*1.46 (0.147)*	*0.01*	−*0.25*	*1.93*
Model 3									
*PGC1A*	*G* carriers	*7.79 (3.22)*	7.4 (4.3)	**57.9 (23.3)**	**21.4 (9.75)**	**2.19 (0.030)**	**0.03**	**10.9**	**31.8**
	non-*G* carriers	*6.20 (3.13)*	6.1 (3.6)	**42.7 (15.5)**	*2.72 (1.41)*	*1.93 (0.055)*	*0.02*	*1.29*	*4.00*

a *A2* allele carriers of *DRD2/ANKK1*: *A2A2* & *A2A1* genotypes, non-*A2* allele carriers: *A1A1* genotype; *D* allele carriers of *ACE*: *DD* & *ID* genotypes, non-*D* allele carriers of *ACE*: II genotype; G allele carriers of *PGC1A: GG & GA* genotypes, non-*G* allele carriers of PGC1A: AA genotype.

b Fonts of dependent variables correspond to fonts of means (s.d.s.) and respective effects.

c All data are adjusted for age and sex.

Multivariate test found that *DRD2/ANKK1* and *ACE*, but not *PGC1A*, allele polymorphism significantly determined the fluctuation of glycemic status in patients with diabetes (Pillai’s traces = 0.06 (0.04), 0.11 (0.07), and 0.01 (0.01); Fs(2) = 3.15 (2.04), 5.82 (3.64), and 0.67 (0.49); *P* = 0.047 (0.135), 0.004 (0.030), and 0.514 (0.617), respectively). HbA_1c_, but not FPG, levels were significantly higher in non-*A2* allele carriers of *DRD2/ANKK1* (*A1A1* genotype) compared with subjects with *A2A2* & *A2A1* genotypes, but both HbA_1c_ and FPG levels were higher in *D* allele carriers of *ACE* (*DD* & *ID* genotypes) compared with subjects with *II* genotype (Table 4).

**Table 4:** Main effects of polymorphisms of the dopamine D2 receptor (*DRD2/ANKK1*), angiotensin 1 converting enzyme (*ACE*), and peroxisome proliferator-activated receptor gamma, coactivator 1 alpha (*PGC1A*) genes on glycemic parameters: fasting plasma glucose (FPG) and glycated hemoglobin A1 (HbA_1c_) in the diabetes group (univariate analyses)

		**Dependent variables**^b^			**Parameter estimates**^c^				
**Independent variables**		FPG (mmol/L)	HbA_1c_ (%)	HbA_1c_ (mmol/mol)	Diabetes group (*N* = 103)				
Genes	Alleles ^a^	M (s.d.)	M (s.d.)	M (s.d.)	B (S.E.)	*t* (*P*)	*η*^2^	Bootstrap 95% CI	
Model 1									
DRD2/ANKK1	*A2* carriers	*9.01 (3.13)*	8.2 (3.9)	**65.8 (19.0)**	**−13.6 (6.1)**	**−2.22 (0.029)**	**0.05**	**−25.0**	**−1.50**
	non-A2 carriers	*9.10 (2.53)*	9.4 (4.0)	**79.4 (20.5)**	**−***0.10 (0.99)*	**−***0.10 (0.921)*	*0.00*	**−***1.84*	*1.51*
Model 2									
ACE	*D* carriers	*9.49 (3.27)*	8.6 (3.9)	**70.3 (19.4)**	**11.7 (4.0)**	**2.89 (0.005)**	**0.08**	**4.22**	**18.61**
	non-*D* carriers	*7.61 (2.25)*	7.5 (3.7)	**58.7 (16.8)**	*1.88 (0.65)*	*2.89 (0.005)*	*0.08*	*0.63*	*3.20*
Model 3									
PGC1A	*G* carriers	*8.95 (3.00)*	8.3 (3.8)	**67.6 (18.4)**	**6.99 (6.97)**	**1.00 (.318)**	**0.01**	**−3.42**	**17.23**
	non-*G* carriers	*9.25 (2.28)*	7.7 (4.2)	**60.6 (22.1)**	**−***0.31 (1.11)*	**−***0.28 (0.781)*	*0.00*	**−***3.29*	*2.65*

a *A2* allele carriers of *DRD2/ANKK1*: *A2A2* & *A2A1* genotypes, non-*A2* allele carriers: *A1A1* genotype; D allele carriers of *ACE*: *DD* & *ID* genotypes, non-*D* allele carriers of *ACE*: II genotype; *G* allele carriers of *PGC1A*: *GG* & *GA* genotypes, non-*G* allele carriers of *PGC1A*: *AA* genotype

b Fonts of dependent variables correspond to the fonts of means (s.d.s.) and respective effects.

c All data are adjusted for age and sex.

The multivariate test found that only the *PGC1A* allele polymorphism determined the fluctuation of glycemic status in the control group (Pillai’s trace = 0.09 (0.10); F(2) = 2.53 (2.89); *P* = 0.089 (0.064)). FPG, but not HbA_1c_, levels were significantly higher in non-*G* allele carriers of *PGC1A* (*AA* genotype) compared with subjects with *GG* & *GA* genotypes (Ms(s.d.s.) = 5.03(0.65) and 4.64(0.49); B(s.e.) = −0.34(0.15), t(P) = −2.21(0.031), η^2^ = 0.08, bootstrap 95% CI: −0.63 to −0.03). No evidence for significant interaction effects on glycemic status was obtained for sex in the main analyses.

## Discussion

Although the distributions of all three genetic loci in the total sample were significantly different from the expectations of HWE, our additional findings attribute this departure to the association of polymorphisms with generations overlapping and the rate of surviving to old age. This is consistent with the genetic models of morbidity and mortality protection effects of *PGC1A* gene *GG* genotype (due to chronic stress resilience effect of high *PGC1A* expression), *ACE I* allele (due to acute stress resilience effect of lower serum ACE activity), and *DRD2/ANKK1* gene polymorphism depending on sex (due to stress resilience effects of dopamine binding in the striatum in females with lower and males with higher sensitivity to motivational/reward factors) (45, 46, 47, 48, 49, 50). The main findings of quantitative associations of the polymorphisms with FPG and HbA_1c_ phenotypes or their lack are independent of these deviations (44).

This study confirmed that fasting plasma glucose and glycated hemoglobin are weakly correlated in diabetes and nondiabetes groups. *DRD2/ANKK1* allele polymorphism determined an uncorrelated part of variations of glycated hemoglobin in the total and clinical groups. This corresponds to the hypothesis that people with high baseline levels of cortical arousal that claim extensive energy delivery for mental processes (e.g., ruminations) are at higher risk for the transition of glucose regulation mechanism from normal to abnormal and for the development of more severe type 2 diabetes (*A1A1* genotype) compared with people with low baseline levels of cortical arousal prone to novelty- or sensation-seeking behaviors (*A2* allele carriers). Indeed *A1A1* genotype determines reduced D2 receptor density and binding, but increased baseline dopamine neuron activity associated behaviorally with high harm avoidance and low novelty seeking behaviors (51). It suggests that if cognitive activity decreases in these people for a long time, the oversupply of glucose is left unclaimed and this increases the risk for diabetes and its higher severity.

In this study, the frequencies of *PGC1A GG* and *GA* genotypes were significantly higher in the diabetes group compared with the control group and this difference was confirmed by the higher glycemic status in general (higher levels of both fasting plasma glucose and glycated hemoglobin) in *PGC1A G-*allele carriers compared with *AA* genotype in total group. Thus, *PGC1A* allele polymorphism determined a correlated part of variations of fasting plasma glucose and glycated hemoglobin in the total group. However, this PGC1A effect on total glycemic status was only related to the risk for the transition of glucose regulation mechanism from normal to abnormal, because PGC1A *GG* and *GA* genotypes determine a lower fasting plasma glucose level uncorrelated with glycated hemoglobin level in a nondiabetic control group. These opposite relationships between *PGC1A* polymorphism and fasting plasma glucose may potentially cancel each other out if assessed in mixed populations (52). This finding corresponds with a potential role of *PGC1A* as a protective factor or a mediator of disease progression depending on affected tissues. *AA* genotype was found to determine reduced activity of *PGC1A* compared with *GG* and *GA* genotypes (53). Thus, higher levels of PGC1A determined by *PGC1A GG* and *GA* genotypes were found to be associated with higher fasting plasma glucose and glycated hemoglobin levels in diabetic patients included in this study. Indeed, it is known that PGC1A activity is strongly activated in the liver and pancreatic *β*-cells of the subjects with obesity and type 2 diabetes (29). This could potentially contribute to increased hepatic glucose production which in turn would contribute to the higher hyperglycemia in patients with type 2 diabetes included in this study. However, overexpression of *PGC1A* in skeletal muscle results in increased glucose uptake by this tissue (29). This can contribute to reducing plasma glucose levels found in nondiabetic subjects with *PGC1A GG* and *GA* genotypes. Thus regularly active muscles due to exercises recommended in the STOP-NIDDM trial become a main protective or resilience factor against the development of insulin resistance and type 2 diabetes only in people with *PGC1A GG* genotype in contrast to those with *PGC1A AA* genotype shown sensitive in the same trial only to glucose-reducing treatment by acarbose (52). It suggests that if muscle activity decreases in the former group of people, the overexpression of PGC1A in the liver and the pancreatic *β*-cells becomes dominant in the effect on plasma glucose level and this increases the risk for diabetes.

Other findings indicated that *ACE* gene deletion polymorphism was associated with the glycemic status indicated by correlated glycated hemoglobin and plasma glucose levels with higher values in *ACE DD* and *ID* genotypes compared with subjects with *II* genotype only in diabetic patients. It corresponds with some reports that found no evidence of *ACE* polymorphism as a risk factor for the development of type 2 diabetes (42), but as a risk factor for more complicated or severe type 2 diabetes (54). The presence of *ACE D* allele determines higher ACE levels and thus higher vascular resistance with decreasing tissue delivery of glucose. The high vascular resistance together with poor glycemic control (i.e., both high plasma glucose and glycated hemoglobin levels) may increases the risk for atherosclerosis (55).

Therefore, the findings suggest that adopting a healthy lifestyle with more intensive cognitive or more intensive physical activity may have a protective (resilience) effect against the development of two different subtypes of type 2 diabetes in susceptible individuals, who are genetically predisposed to more intensive energy supply for respective brain or muscle activity. The interventions for increasing cognitive activity or decreasing vascular resistance may further protect against complications in patients with these two cortical or metabolic/vascular subtypes of type 2 diabetes. This further suggests that the metabolic/vascular pathway in the development of diabetes and its severity may be associated with ‘lazy’ muscles determining a concordant increase in glycated hemoglobin and fasting plasma glucose levels. ‘Cortical arousal’ mechanism of the development of type 2 diabetes may be associated with ‘idled’ brain determining an isolated increase in glycated hemoglobin level without a significant effect on fasting plasma glucose. Thus, both glycated hemoglobin and fasting plasma glucose should be evaluated to indicate principal pathogenetic differences in the development of type 2 diabetes for its correct treatment and prevention.

The main limitation of this study is the relatively small sample size. However, according to the power analysis, larger sample sizes would be relevant in the case of gene polymorphisms for which effects were not expected to show evidence of practical importance or statistical significance to avoid type II (‘false-negative’) errors. The study’s results confirm that the present sample size had enough power to demonstrate the predicted effects in order to support the main hypothesis. However, further validation of these findings in a larger population involving other factors modifying these and other related gene expressions is required. Modification of detected significant effects by variations in glycemic control and DNA, mRNA and protein expressions as well as polymorphisms of other genes with the same effects on glycated hemoglobin and fasting plasma glucose levels can explain weak effect sizes obtained in the study. This study was conducted to obtain proof-of-concept where genome-wide association research is methodologically ineffective. Future research should support the concept with respect to other genetic, epigenetic, and proteomic factors. These factors should increase explained variance or effect size of both pathogenic mechanisms and thus demonstrate clinical importance of the two subtypes of type 2 diabetes and that both glycemic parameters are not interchangeable as indicators since they contribute unique information in its pathogenesis. This consideration should hold expert committees in diabetes from precipitate revisions of the diagnostic criteria for diabetes in favor of either indicator as more reliable (2). Clinical trials would demonstrate a beneficial effect of different cognitively or physically oriented lifestyles in individuals with different susceptibility to hyperglycemic effects of the decrease in brain or muscle activity.

## Declaration of interest

The authors declare that there is no conflict of interest that could be perceived as prejudicing the impartiality of the research reported.

## Funding

The study was supported by the Russian Ministry of Science and Education project No. 2934 led by M.K.N.

## Author contribution statement

MKN designed and coordinated the study and reviewed/edited the manuscript, DMD conceived the study, researched data, contributed to the design and coordination, and wrote the manuscript. Both authors read and approved the final version.

## Accession code

Phenotype and genotype data of the study have been deposited at the European Genome-phenome Archive (EGA, http://www.ebi.ac.uk/ega/), which is hosted by the EBI, under DAC accession number EGAC00001000337.
